# Unpacking the ‘black box of horrendousness’: a qualitative exploration of the barriers and facilitators to conducting trials involving adults lacking capacity to consent

**DOI:** 10.1186/s13063-022-06422-6

**Published:** 2022-06-06

**Authors:** Victoria Shepherd, Kerenza Hood, Fiona Wood

**Affiliations:** 1 Centre for Trials Research, Cardiff University, Cardiff, UK; 2 PRIME Centre Wales, Cardiff, UK; 3 Division of Population Medicine, Cardiff University, Cardiff, UK

**Keywords:** Informed consent, Mental capacity, Randomised controlled trials, Inclusion, Under-served populations, Qualitative

## Abstract

**Background:**

Trials involving adults who lack capacity to consent encounter a range of ethical and methodological challenges, resulting in these populations frequently being excluded from research. Currently, there is little evidence regarding the nature and extent of these challenges, nor strategies to improve the design and conduct of such trials. This qualitative study explored researchers’ and healthcare professionals’ experiences of the barriers and facilitators to conducting trials involving adults lacking capacity to consent.

**Methods:**

Semi-structured interviews were conducted remotely with 26 researchers and healthcare professionals with experience in a range of roles, trial populations and settings across the UK. Data were analysed using thematic analysis.

**Results:**

A number of inter-related barriers and facilitators were identified and mapped against key trial processes including during trial design decisions, navigating ethical approval, assessing capacity, identifying and involving alternative decision-makers and when revisiting consent. Three themes were identified: (1) the perceived and actual complexity of trials involving adults lacking capacity, (2) importance of having access to appropriate support and resources and (3) need for building greater knowledge and expertise to support future trials. Barriers to trials included the complexity of the legal frameworks, the role of gatekeepers, a lack of access to expertise and training, and the resource-intensive nature of these trials. The ability to conduct trials was facilitated by having prior experience with these populations, effective communication between research teams, public involvement contributions, and the availability of additional data to inform the trial. Participants also identified a range of context-specific recruitment issues and highlighted the importance of ‘designing in’ flexibility and the use of adaptive strategies which were especially important for trials during the COVID-19 pandemic. Participants identified a need for better training and support.

**Conclusions:**

Researchers encountered a number of barriers, including both generic and context or population-specific challenges, which may be reinforced by wider factors such as resource limitations and knowledge deficits. Greater access to expertise and training, and the development of supportive interventions and tailored guidance, is urgently needed in order to build research capacity in this area and facilitate the successful delivery of trials involving this under-served population.

## Background

An estimated two million people in England and Wales have significantly impaired decision-making, which may be due to acute medical events, or through long-term conditions affecting cognitive function such as dementia and mental illness, or associated with learning disabilities or at the end of life [1]. Up to half of patients in acute medical and psychiatric healthcare settings lack decision-making capacity [2, 3], and this rises to around 70% in settings such as care homes [4] and 90% in critical care [5]. Research into conditions affecting these groups, who often experience higher care needs and require greater health resource use [6], is vital. However, adults with impaired decision-making and who therefore have an impaired ability to provide their own consent, are often excluded from research [7–9]. This exclusion is a result of the complex ethical and practical challenges encountered when seeking to include adults who lack capacity in research and the daunting range of methodological, structural and systemic barriers to their inclusion [10].

### Impact of exclusion from trials for this under-served population

Despite a growing emphasis on making trials more inclusive to under-represented or under-served populations, such as the National Institute for Health and Care Research (NIHR) INCLUDE initiative [11], few trials are designed to include participants who lack capacity, and the numbers of participants unable to consent who are actually recruited are worryingly low [12]. This exclusion is particularly concerning in conditions where the prevalence of cognitive impairment is high. For example, 1 in 3 patients with a hip fractures have concomitant cognitive impairment, yet this population is excluded or ignored in 8 out of 10 hip fracture trials [13]. Consent-based recruitment biases have a clear impact on the generalisability of trials, as demonstrated in a trial in acute haemorrhagic shock (CONTROL) where the consent model used reduced enrolment by 80–90% in the USA and led to a trial population that was not representative of the intended target population and the trial being halted due to futility [14]. This widespread exclusion of adults lacking capacity results in a poorer evidence base for their treatment and care compared to other groups, leading to ‘evidence *biased*’ care [15] and contributing to the health inequalities many already experience [16].

COVID-19 has shone a spotlight on this issue through the disproportionate impact on populations who are under-served by research, many of whom have impaired capacity to consent. Dementia is an age-independent risk factor for contracting COVID-19, subsequently requiring hospitalisation, and death [17], with older people living in care homes at particularly high risk [18]. However, a review of COVID-19 trials found that half excluded older people, with other relevant indirect exclusions such as cognitive impairment [19]. Older people were excluded from all of the vaccine trials included in the review [19]. This has led to criticisms that, despite the disproportionate impact on people living in care homes, the research community has largely ignored this population when conducting clinical research on COVID-19 [20]. Similarly, people with learning disabilities with COVID-19 were five times more likely to be admitted to hospital and eight times more likely to die than people without a learning disability [21]. Yet, prior to the pandemic, 90% of RCTs were designed in a way that excludes people with a learning disability [7].

### Ethical and legal complexities of trials including adults who lack capacity to consent

Trials involving adults with impaired capacity to consent are recognised as being ethically and legally complex, with the need to ensure that special safeguards are in place to protect the interests of this group [22]. The legal arrangements for including adults who lack capacity vary by jurisdiction and the type of research in question. In the UK, clinical trials of investigational medicinal products (CTIMPs) are governed by the Medicines for Human Use (Clinical Trial) Regulations which permits a legal representative to give consent on behalf of an adult who lacks capacity [23]. However, other types of research (including trials not classified as CTIMPs) are governed by mental capacity legislation with different laws in place across the UK [15]. There are important differences between how non-CTIMPs involving adults who lack capacity, particularly in emergency situations, are governed. For example, where the treatment needs to be given as a matter of urgency and it is not possible to obtain either the participant’s consent or to consult a family member or carer before participation, in England and Wales the Mental Capacity Act (MCA) permits recruitment into the study if it is done in accordance with a protocol previously approved by a research ethics committee (REC) [24]. However, in Scotland, there are no similar provisions under the Adults with Incapacity (Scotland) Act (AWI) [25] and so such research cannot be lawfully carried out in Scotland [22]. Unsurprisingly, these complexities have resulted in misunderstandings about the legal frameworks and how they apply to research involving adults lacking capacity [26], which then translates into incomplete and misinterpreted information provided to legal representatives and consultees [27]. This in turn contributes to the decisional and emotional burden experienced by family members acting as legal representatives and consultees [28].

In the UK, other safeguards designed to protect this group considered ‘vulnerable’ include the requirement for the research to be approved by an appropriate body, which is one of the panel of national RECs that are flagged to review studies involving adults unable to consent for themselves [29]. Trials in populations, settings and conditions where capacity to consent is of particular relevance require careful design, planning and implementation to incorporate these important safeguards whilst ensuring that these populations have the opportunity to contribute to and benefit from research.

### Importance of understanding and addressing the barriers to inclusion

Strategies to improve the conduct of trials involving adults who lack capacity will not be effective unless the barriers are recognised and addressed. In recent years, there has been a huge focus on improving trial conduct through initiatives such as Trial Forge [30] and by the MRC-NIHR Trials Methodology Research Partnership (formerly Methodology Hubs) [31] in the UK, and the Clinical Trials Transformation Initiative in the US [32]. Considerable work has also been undertaken to identify and address the priority areas for research into recruitment and retention in trials [33]. However, these ambitious programmes have almost exclusively concerned trials involving adults who provide their own consent to research. Given the considerable additional complexities in trials involving adults who are unable to provide consent, similarly large-scale work is needed to ensure that these populations are better served by research.

This qualitative study marks the first step to empirically explore the barriers and facilitators to conducting trials with adults who lack capacity to consent in the UK across a range of populations and settings, and to identify potential areas for future research and interventions to support their inclusion. The objectives of the study were to explore researchers’ and healthcare professionals’ views about the barriers and facilitators to research involving adults lacking capacity to consent and to establish their training, education and support needs for designing and conducting research with these populations.

## Methods

We conducted remote semi-structured interviews with researchers in health and social care and healthcare professionals who design and conduct research with adults who lack capacity.

### Recruitment

Participants were recruited through UK-wide research networks (e.g. MRC-NIHR TMRP, UK Trial Managers’ Network (UKTMN)) who disseminated information about the study to their members, and via social media (e.g. Twitter). The aim of the interviews was to obtain a range of views and experiences, and so maximum variation sampling was used to ensure the inclusion of participants with diverse roles and experiences. This heterogeneity enabled an understanding of variations in experiences whilst also investigating core elements and shared issues [34].

Participants were iteratively selected to take part in an interview based on criteria used to construct the sample frame such as their role (e.g. Trial Managers, Research Nurses, Chief Investigators and other members of research team), experience with populations and conditions (e.g. dementia, frailty, COVID, trauma, learning disability, stroke), research settings (e.g. ICU, acute care, community, care homes), types of study/intervention (e.g. CTIMPs, surgery, complex intervention) and stage of the trial (e.g. completed, recently commenced, requiring adaptions during COVID-19). As this is the first study in this area, the lack of a clear understanding about the range of experiences meant that an iterative approach to sampling was warranted, where analysis of initial data influenced subsequent recruitment decisions [34]. Eligibility was assessed by email discussion with potential participants about their role and experiences, and eligible participants were then invited to take part in a single one-to-one interview by telephone or online (via Zoom video conferencing).

### Ethical considerations

This study was reviewed by the Cardiff University School of Medicine Research Ethics Committee (SMREC ref. 21.28) and received a favourable opinion. Prior to agreeing to take part in an interview, participants were provided with a participant information sheet and consent form by email and given the opportunity to ask questions. All study participants provided verbal consent prior to participation which was audio recorded. This approach is in accordance with the Health Research Authority (HRA) guidance that consent may be obtained orally (or by any other means of communication) for research studies that are not clinical trials [35]. Participants were allocated a unique study ID to ensure anonymity of participants, and all trial names and other identifying features were removed from transcripts prior to analysis to ensure anonymity of the trials discussed.

### Data collection

The topic guide for the interviews was developed by the research team and was informed by previous research [10]. It was piloted with two researchers with similar roles to participants. The guide was used to help structure the interview, although the exact phrasing of questions and sequence of areas of enquiry was not intended to be rigidly adhered to and participants were openly encouraged to talk about other areas and experiences that they felt were relevant to the topic. Notes were made during the interviews to highlight significant points for further discussion and researcher observations were recorded in order to capture any contextual data. Participants were also provided with information about a decision support tool that has been developed by the research team [36] and asked for their views about conducting a trial to establish its effectiveness using SWAT (Study Within a Trial) methodology [37], and how it might be implemented in practice. Data relating to this topic area are not included in this paper but will be reported separately.

Data collection took place between April and June 2021. Recruitment and data collection continued until the research team considered that sufficient data (defined as the depth, diversity, and adequacy of the data) were obtained to answer the research questions [38]. A total of 26 interviews were conducted with researchers and healthcare professionals, of which 25 interviews were conducted by video conferencing (Zoom) and one by telephone. All interviews were conducted by the first author, who is female and a nurse by profession and is experienced in conducting qualitative research. The interviews were digitally audiorecorded with consent. The mean duration of the interviews was 45 min (range 34–56 min).

### Data analysis

Interviews were transcribed verbatim by an external professional transcription service who have previously been used by the research team. The transcripts were checked by the first author for accuracy and completeness against the source data and anonymised. Thematic analysis of the interviews was undertaken through a process of familiarisation with the data, data coding, generation of initial themes, and refining the themes [39]. Qualitative data analysis software (NVivo 12, QRS International) was used to support data analysis. Transcripts were read and re-read by the first author to achieve familiarity, and data were iteratively coded using codes derived from the data, and those identified a priori. Data generation and analysis was undertaken concurrently to facilitate an iterative approach to coding and identification of early initial themes. Analysis of the first four interview transcripts was undertaken by the first author, and then discussed with the wider research team to review the coding framework and coding process. The remaining transcripts were then iteratively coded by the first author with a second researcher (a medical sociologist with extensive experience in qualitative research) reviewing the coding of a subset of transcripts (*n*=4), selected to represent a range of different researcher roles, to enable greater critical reflexivity in the analytical process [40] and ensure coding validity. In accordance with reflexive thematic analysis, a statistical ‘inter-rater reliability’ approach to coding validity was not used [41]. Initial themes were generated by the first author and then developed and refined through an iterative process of discussions about data interpretation by the research team, before then finalising these themes.

## Results

Participants consisted of four Chief Investigators (CI), ten Trial Managers (TM) or Senior Trial Managers (STM), four Research Nurse/Practitioners or Senior Research Nurses and two non-clinical researchers who recruited participants, two qualitative researchers and four who had other roles such as a CTU manager (see Table 1). In terms of trial settings, their experiences ranged from trials in acute or emergency settings such as critical care, pre-hospital, and emergency departments (ED), to primary care and care homes. Trial populations included people living with long-term conditions such as dementia and mental health conditions, people receiving palliative care and people with learning disabilities, as well as acute events such as trauma, stroke, cardiac arrest and myocardial infarction. Trial interventions included a range of CTIMPs, as well as non-CTIMPs including complex interventions and surgical trials. Due to the timing of the interviews (mid 2021), many participants had recently experienced designing and conducting COVID-19 trials or reported their experiences of the impact of COVID-19 on trials which had been designed and set up pre-pandemic.

**Table 1:** Participant characteristics

	No. of participants (***n***=26)
**Role**	
Chief Investigator/Principal Investigator	4
Trial Manager/Project Manager	4
Senior Trial Manager/Programme Manager	6
Research Nurse/Research Practitioner	2
Senior Research Nurse/Team Lead	2
Qualitative Researcher	2
Research Associate/Senior Research Associate (recruiting participants)	2
Other (e.g. statistician, Clinical Trials Unit manager, research governance lead)	4
**Experience in role**	
0–4 years	6
5–9 years	10
10+ years	10
**Location**	
England	24
Scotland	2

Participants described a wide range of experiences with previous and ongoing trials involving adults who lack capacity to consent and reported how a number of factors influenced various key processes throughout the design, conduct and delivery of a trial (see Table 2). These processes can be categorised as making trial design decisions, navigating ethical approval for the trial, providing information to and supporting decision-making by potential participants with impaired capacity, undertaking assessment of capacity to consent, the involvement of alternative decision-makers (consultees and legal representatives), and revisiting consent and consultation throughout the trial.

**Table 2:** Key processes in the the design, conduct and delivery of trials involving adults with impaired capacity

Process	Description of process
Making trial design decisions	Decisions about the design of the trial made prospectively (e.g. eligibility criteria) and during the ongoing conduct of the trial (e.g. adaptations following a feasibility study)
Navigating ethical approval	Process of seeking review by a Research Ethics Committee and the challenges of obtaining a favourable opinion. May involve multiple approvals process across the UK nations and beyond for multi-centre multi-national trials
Informing and supporting the participant	Providing information about the trial to a potential participant with a communication disability and/or impaired capacity, and supporting their involvement in making a decision about participation
Assessment of capacity to consent	Assessment of a person’s mental capacity with respect to their ability to make a decision about participating in a trial, in accordance with the process set out in the Mental Capacity Act 2005 or other devolved legislation
Involvement of alternative decision-maker	Process of identifying and consulting an alternative decision-maker (e.g. consultee or legal representative) on behalf of a participant who lacks capacity to consent. In accordance with the legal frameworks, this may involve someone acting in either a personal or professional capacity
Revisiting consent and consultation	Informed consent is an ongoing process which may require revisiting consent and/or consultation with alternative decision-makers due to changes in the participant’s capacity during the trial. This includes in emergency research where a participant may have been recruited without prior consent or consultation

**Fig. 1:**
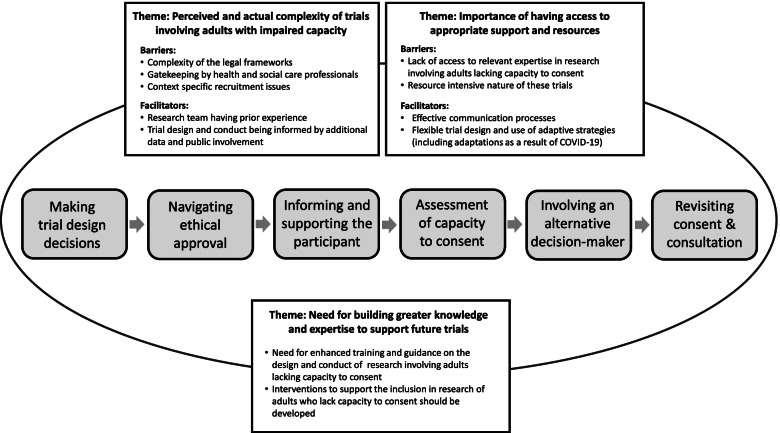
Diagram of the barriers and facilitators to conducting trials involving adults lacking capacity to consent

Participants described a number of inter-related barriers and facilitators, of which the main contributors are outlined below using anonymised illustrative quotations and are summarised in Table 3 below. Three over-arching themes were identified: (1) the perceived and actual complexity of trials involving adults with impaired capacity, (2) the importance of having access to support and resources and (3) the need for building a body of knowledge and expertise to support future trials. The diagram in Fig. 1 shows the themes and related barriers and facilitators.

**Table 3:** Summary of barriers and facilitators to conducting trials involving adults with impaired capacity

Process	Barriers	Facilitators
Making trial design decisions	∙ Complexity of the legal frameworks governing trials involving adults lacking capacity ∙ Lack of access to relevant expertise in trial teams ∙ Lack of effective training and information about conducting trials with adults who lack capacity ∙ Context-specific recruitment issues (e.g. lack of consultees/legal representative in some settings, role of gatekeepers) ∙ Pressure of recruitment targets and timescales which do not take account of the additional time required to recruit adults lacking capacity ∙ Lack of resources (e.g. research nurse time) to meet the additional resources required ∙ Lack of appropriate and agreed outcome measures for participants with cognitive impairment and their proxies	∙ Experience and familiarity with trials with adults lacking capacity to consent ∙ Availability of additional data (qualitative, feasibility) to inform the design and conduct of the trial ∙ Flexibility and use of adaptive strategies (e.g. allowing remote contact and consent from consultees and legal representatives) ∙ Feedback from funders and reviewers which recognises the importance of the inclusion of adults lacking capacity and supports their inclusion
Navigating ethical approval	∙ Lack of knowledge and understanding by RECs and research teams about the legal provisions for adults lacking capacity and how they are implemented in practice ∙ Differences in the legal provisions between nations (e.g. between England & Wales and Scotland) and the impact of dual applications and multiple sets of trial documents ∙ Lack of provision for conducting emergency non-CTIMP research in Scotland under AWI ∙ Inconsistency in REC reviews (both between and within RECs) and inaccuracies in advice/requirements	∙ Having an established relationship with a REC who have previously reviewed trials involving adults who lack capacity by the research team ∙ Effective communication with RECs who are able to offer knowledgeable advice to trial teams
Informing and supporting the participant	∙ Complex and lengthy Participant Information Sheets that are not cognitively or linguistically accessible ∙ Lack of access to timely translation services that are appropriate for people with cognitive impairment who do not have English as a first language	∙ Availability of accessible trial information (e.g. easy-read, pictorial, brief summary version) ∙ Involvement of family and carers to support the person with cognitive impairment to make a decision about research participation
Assessment of capacity to consent	∙ Requirement to conduct assess capacity remotely which is more challenging and unfamiliar ∙ Lack of access to background information held in clinical records	∙ Seeking the views of others who are familiar with the person (e.g. family, usual carers) ∙ Access to expertise on communication and assessing capacity (e.g. Speech & Language Therapist)
Involvement of alternative decision-maker	∙ Reliance on care team to identify and approach family members ∙ Gatekeeping or lack of engagement from families and care team ∙ Uncertainty around the legal and ethical role of alternative decision-makers ∙ Challenging process of decision-making when the person’s wishes and preferences are unknown	∙ Flexible consent arrangements (e.g. telephone consultation, verbal consent/agreement) ∙ Established processes for involving alternative decision-makers (e.g. prospective list of care team members able and willing to act as consultee/legal representative)
Revisiting consent and consultation	∙ Participant is discharged, transferred or dies before consent can be sought (e.g. deferred consent or recovered capacity) ∙ Reliance on remote contact only during a trial (e.g. postal follow-up only)	∙ Regular communication and establishing a rapport with the participant and their care team ∙ Prospective planning for changes in capacity (e.g. prospectively obtaining family contact details, ongoing involvement of family/carer)

Key: *REC* research ethics committee, *CTIMP* clinical trial of an investigational medicinal product, *AWI* Adults with Incapacity (Scotland) Act 2000

### Perceived and actual complexity of trials involving adults with impaired capacity

#### Complexity of the legal frameworks

Participants’ experiences of the complexity of the legal frameworks governing trials involving adults lacking capacity, and the perceived complexity by those without direct experience, acted as a barrier to the design and conduct of trials. This included the difference between the frameworks governing CTIMP and non-CTIMP studies, the implications for who could be involved as an alternative decision-maker, the legal basis for their decision, and the different terminology used. However, having experience across different types of trials reduced the complexity for some researchers.

It is quite complicated because the rules change depending on whether it’s a drug trial or whatever. There’s consultees or legal representatives and they’re slightly different even though they’re more or less the same thing. The legalities of it are quite complex but I think once you’ve got your head around it, because I see it all the time and I do it all the time it’s relatively easy for me, whereas for a researcher who’s maybe doing this for the first time, it’s not so much. [ID 01, research governance lead].

Participants described trials involving adults lacking capacity as a minefield, and many expressed fears that consent might be obtained from the ‘wrong person’. This trepidation was a particular concern when research staff had less experience in research with adults lacking capacity, where family circumstances were not straightforward, where professionals were acting as a consultee or legal representative, or where consent pathways were more complex. These fears centred around the legal and professional implications of unintentional non-compliance with the legal frameworks. For some, this impacted on how trial design decisions were made in relation to selecting eligibility criteria which led to the subsequent exclusion of adults lacking capacity such as those with learning disabilities or dementia as the safest default position. Where this occurred, participants acknowledged the problematic nature of this exclusion.

As a researcher it feels like just an insurmountable black box of horrendousness that I dare not go [into]. It feels very much [that] if you get this wrong you will be illegal … and the ethics police will come for you or something. So, we just try and avoid it and then I’m not really sure that that’s the right thing. It’s scary. [ID 02, care home researcher]

The differing legal frameworks across the UK, EU and beyond acted as a barrier to conducting multi-centre trials. This was particularly encountered in emergency non-CTIMP research which is not currently permitted in Scotland, and so trials were either unable to include Scottish sites or they had to amend the trial design to have different recruitment pathways in Scotland which aligned with the Adults with Incapacity (Scotland) Act [25]. Other participants reported that their trials that did not reach the definition of being emergency research but were ‘borderline’ cases with narrow recruitment windows or were being conducted in emergency settings, and so also encountered problems through being on the legal ‘cusp’. This demonstrated that the legal dichotomy that exists between emergency and non-emergency research was much less clearly defined in practice.

The complexity surrounding the legal frameworks also contributed to the challenges of navigating ethics review for these trials. Participants reported it being often a difficult process and sometimes a ‘brutal’ or ‘baptism of fire’ experience. This included situations where RECs raised concerns about the processes being proposed, although researchers considered that they had adhered to the legal frameworks to the best of their ability to interpret the requirements. REC concerns sometimes centred on who would act as a consultee or legal representative, particularly where they had a dual role as a carer and so potentially may indirectly benefit from the intervention.

The REC had a problem with that consultee being the carer of the individual because they felt that consultee advice wouldn’t be that impartial and we included data collection from carers. So, we had to try and separate that out, and that’s quite difficult because when you’re looking for a consultee, the nearest person to that individual is likely to be their carer and that is the person that is likely to know them best. So, it’s a bit of a double-edged sword, isn’t it? [ID 04, research programme manager]

Participants also reported inconsistencies in ethics reviews, with conflicting advice being provided both between different RECs and from the same REC regarding different studies. Some participants reported receiving inaccurate advice from RECs which they felt obliged to follow in order to obtain the favourable opinion that they needed, despite knowing the information to be inconsistent with the legal frameworks.

I remember myself and the project lead going to the ethics meeting and being grilled about who would take on the role of the personal consultee and [the REC] being adamant that it needed to be the lasting power of attorney for health and welfare, which it doesn’t need to be, which I did try to say but they weren’t really having any of it. So, [we] dutifully we put in the protocol that we have to firstly approach a lasting power of attorney if there is one in place, but if not or if not suitable then we will speak to another member of the family or another friend. So, we’ve kind of had to follow that approach which feels inappropriate really but that was imposed by ethics. [ID 06, trial manager]

There were particular challenges when navigating ethical approval for emergency research conducted without prior consent or consultation (often termed deferred consent). This was illustrated in a pre-hospital cardiac arrest trial where questions around the ability of members of the public to ‘opt out’ of the trial were raised by the REC, although this could potentially lead to delays in administering life-saving treatment.

They thought it was important that people could let us know that they didn’t want to be in the trial, and I think that it was a misguided piece of advice. So, we did it and we opened recruitment and no one got in contact with us to say, ‘can you please put me on the register?’ because nobody knows when someone might go into cardiac arrest. So, I think it’s a burden on the researchers, without any really meaningful outcome. If you check the peoples’ name against some register and say there were a hundred names on the register, it means the paramedics are going to have to pause while they do recruitment to assess if they should recruit, randomise this person. It’s not safe and makes the research unethical. [ID 23, senior trial manager]

Research nurses were less likely to share trial teams’ concerns about the complexity of the legal frameworks or the need for more information. They were generally satisfied that they were complying with the legal frameworks if they complied with the approach that was stated in the protocol. This was partly influenced by their perception about where responsibilities lay, but also because they worked across multiple trials to recruit a range of participants in high pressure environments and so needed straightforward processes to follow.

We are all assuming, rightly or wrongly, that these things have been dealt with by the MHRA and all the legal groups. I think it’s something that needs to be handled by the trial centre … we do need to query these things, but I think if it was all done at the beginning, and it was all set up and we don’t have to worry about it and there was a document that came in the introduction part of the trial, then that’s as much as we would need to know in my opinion. [ID 24, research nurse]

#### Benefit of having prior experience

Participants frequently reported how members of the team having relevant prior experience acted as a facilitator. This included having clinically trained researchers who were familiar with cognitive impairment and issues around capacity either as investigators on the trial or located within the Clinical Trials Unit (CTU), as well as CTU staff who had prior experience with conducting trials involving adults who lacked capacity. However, while some participants were members of teams or located within centres with considerable expertise in this area, many reported a more ad hoc nature of having access to people with relevant experience due to the relative rarity of such trials. Of particular value was their practical experience of the application of the legal frameworks and an understanding of mental capacity assessment processes.

Not a lot of studies that come through even our unit have had to go down the route of dealing with capacity and so forth. But, actually, although you’re aware of regulations, it’s never until you really need to apply them that you start to learn the ‘ins and outs’, is it? [ID 22, senior trial manager]

Having experience within trials teams also increased their confidence in designing trials to include adults who lacked capacity, partly due to a lack of information and support available from elsewhere.

It was really difficult to get anything concrete that was useful. And that’s where my colleague would come in because I would have been very reluctant to have proposed this without [their] expertise of being an ex-GP. [ID 26, Chief Investigator]

Research nurses also described the benefit of having experience in the same clinical area or population that they were conducting trials in, which increased their confidence in recruiting participants who lacked capacity. Where research nurses lacked personal experience, they drew on other team members’ experience, and having a team with a mixture of clinical backgrounds was seen as advantageous.

#### Gatekeeping by health and social care professionals

In addition to the role gatekeepers can play in research generally, participants described the additional layer of gatekeeping that can occur for people with cognitive impairment in a wide range of settings including in mental health, older people and in care homes.

We have a lot of care coordinators who go oh no, my patients are just not well enough, they wouldn’t be interested in that, they’re not well enough, you know, it’s not something that would be appropriate to talk to them about. [ID 01, research governance lead]

I think consent and capacity to consent in care homes is quite a tricky issue, because you do have these gatekeepers along the way as well. They’ve got another layer with the care home staff there, that’s quite difficult. [ID 04, research programme manager]

In some situations, participants reported how the involvement of alternative agencies introduced additional gatekeeping barriers, especially when these groups would not be considered to hold any decision-making authority according to the legal frameworks. This sometimes led to considerable delays.

You identify that someone might be eligible, and you want to get them involved, and not spend months and months contacting every relative. And, we did have a few cases [when] we needed to have a social services meeting to decide whether the person can take part. Some of them went on about two or three months, and then the nurses are just waiting for a decision on how to move forward with the patient. [ID 20, senior trial manager]

Gatekeeping was perceived by participants as being paternalistic, recognising that health and social care staff intended to protect those in their care from the perceived burdens and harms that they associated with research. Participants suggested that further work to integrate care and research was needed, and more education and awareness about research and about the importance and justification to include people with impaired capacity in research in order to generate the evidence to inform their care.

#### Trial design and conduct being informed by additional data and public involvement

The importance of having access to additional data when designing and conducting trials involving adults with impaired capacity was highlighted by a number of researchers. This included feasibility studies which provided estimates of the number of potential participants who might lack capacity, allowed the feasibility and acceptability of consent models to be assessed, and enabled processes for identifying and seeking agreement or consent from alternative decision-makers to be explored. It also enabled trial teams to understand how often there might not be a family member to act as a consultee or legal representative for participants who would therefore require a nominated consultee or professional legal representative to be identified instead. Data collection processes and completion rates could also be assessed, including where proxy-reported data were being provided by carers or family members, which could then be amended for the main trial if need be.

Some participants described the value of having qualitative data to directly inform the design and conduct of a trial. This was gained through either a qualitative study being embedded during a feasibility study or in the main trial, or formed part of a process evaluation. In some cases, qualitative interviews exploring participants’, clinicians, and carers’ experiences of recruitment raised unanticipated concerns about participants’ capacity to consent at the time of enrolment which led to these issues being raised with trial managers and clinical staff involved in recruitment. Providing this feedback synchronously, rather than waiting for the formal qualitative analysis that followed, provided an opportunity to revise consent arrangements and revisit site staff training on capacity and consent in real time.

Qualitative research in the context of the trials highlights issues that you wouldn’t find if you didn’t do it. Because we would have taken that consent and assumed everything was fine and it’s only because I did those interviews and I had that relationship with [the trial manager], that we were able to kind of iron out some of these things and try and do something about it. Otherwise, I think we would have just assumed all was okay. [ID 12, qualitative researcher]

Researchers who had experienced the value of concurrent qualitative research described how they would purposefully design future trials in these populations to include qualitative research focusing on aspects of consent and capacity during trial recruitment, with early findings reported to inform trial conduct.

Public involvement was also reported to have a facilitative effect through a number of different mechanisms. At the early design and funding application stages, lay research partner support for including people with impaired capacity was seen as crucial for the trial. During the ethics review process, it strengthened the ethical justification for their inclusion, and also ensured that trial materials were appropriately worded and accessibly designed for both people with cognitive impairment and their families.

Our Chair for that [public involvement] group was a stroke survivor and they were also involved in the Ethics application and went to the Ethics Committee …. I think that certainly was helpful for [the REC members] to know that we’ve involved patients in not only the patient facing documentation, but also in the discussions involving people without capacity and yes, they would have wanted to be involved, and believed [their spouse] would have made that decision for them if they had been asked. [ID 21, trial manager]

#### Context-specific recruitment issues

Whilst many of the barriers and facilitators were generic across trials and populations, a number of participants reported context-specific issues. This included the impact from a lack of research staff in less research active areas such as community-based services, or where clinical and research roles had to be combined such as paramedics attending a call, or settings outside the NHS such as care homes. Even areas with high research activity such as primary care encountered challenges recruiting people with impaired capacity as they often relied on recruitment methods such as screening patient records and sending out invitations which these populations are unlikely to be able to respond to. Clinical pathways which involved transition between care settings such as discharge from hospital to community follow-up, often encountered challenges around maintaining contact and continuity of data collection with patients who were unable to self-report.

Where trials used postal recruitment and data collection methods, there were particular challenges around interpreting non-responses or assessing capacity. It was also difficult to recruit people with fluctuating or borderline capacity who lived alone but required consultee or legal representative involvement—or which may be required during the duration of the trial if capacity were to be lost.

Quite often our participants live independently at home or semi independently. It's quite difficult to track down a carer in a lot of instances so even trying to get hold of a consultee is quite difficult for the researchers. First of all, you have to get the potential participant to name somebody and then getting the contact information for them and then trying to track them down has got issues of its own. [ID 04, research programme manager]

Depending on the nature of the intervention, some trials tried to overcome these challenges by sending the initial recruitment invitation addressed to the household, or including a statement in the initial invitation or questionnaire in which the person could indicate that they had problems with their memory or understanding, or they recruited through registries such as NIHR Join Dementia Research [42] which includes an option for a person’s representative to be contacted about a study.

Trials that recruited care home residents who lacked capacity encountered specific challenges around relying on busy care home staff to assist with determining eligibility, approaching care home residents on behalf of the researchers and informing mental capacity assessments. They also played a key role in contacting family members to act as a consultee or legal representative, data collection, monitoring and reporting changes in residents’ condition (including capacity to provide ongoing consent) and potentially delivering the intervention and reporting compliance/adherence. In addition to their gatekeeping role, these additional roles added to the complexity of trials in care homes as the staff may lack the time or motivation to take on these additional roles on top of an already heavy workload.

In other contexts, trials with short time windows for recruitment were very challenging as they required a compressed process for capacity assessment and identification of alternative decision-maker if required. This included trials in ED and ICU settings, as well as those involving trauma where there were additional pressures of recruiting within expected surgical timeframes against the backdrop of operating list timings. These trials might also involve randomisation to surgical or non-surgical management where clinician equipoise and patient/family preferences could strongly influence recruitment. There were also additional challenges around surgeons’ views about the requirements for capacity to consent to research as opposed to their usual consent practices for treatment decisions, and where cognitive impairment associated with the traumatic event such as a hip fracture following a fall may be superimposed on existing cognitive issues due to a condition such as dementia.

The argument that we often got back was, “Well, this isn’t different to what we were doing with routine practice.” But our start has always been, “But it isn’t the same as routine practice, because you’re asking someone to take part in a trial.” [ID 12, qualitative researcher]

Another important context was the nature of the intervention and whether it was designed to enable participants with cognitive impairment to engage with it. This was particularly challenging when potential participants might have physical disabilities or hearing and vision impairments in addition to cognitive and or communication impairment, for example in conditions such as a stroke. Additional linguistic complexities may also arise if English is not the person’s first language. These issues were often difficult to untangle and were sometimes conflated by researchers, trial recruiters and RECs. Participants highlighted the importance of exploring the feasibility of implementing the intervention in these different contexts, in addition to the feasibility of the consent and consultation arrangements.

Issues around data collection and the lack of validated questionnaires for these populations was also cited as a barrier to conducting trials, as well as a lack of consensus around which outcome measures should be used. Some researchers described the additional challenges this would bring in terms of the impact on data analysis.

There was definitely a lot of thought in that trial about which questionnaires were appropriate to be completed by a proxy, if there were validated proxy versions of those questionnaires, and if there weren’t, what was the most appropriate wording for us to use? There were questionnaires that we did remove because we thought that it wasn’t really appropriate for someone else to answer that on their behalf. That will definitely have an impact in the analysis side of things, because we’ll have a lot of missing data on those. [ID 05, trial statistician]

In one study, rather than using formal interviews to collect qualitative data with care home residents with cognitive impairment, the researchers reported the use of ‘in the moment conversations’ to capture their views and feelings about the intervention. These conversations would be recorded in the form of written notes by the researchers, rather than being audio or video recorded. This was seen as a pragmatic way of including the voices of a population which has high levels of cognitive impairment where formal interviews were unlikely to provide in-depth or ‘quality’ data.

Research teams also described the importance of recording and reporting the use of supportive and adaptive strategies in different contexts, such as including a question about whether participants had researcher support to complete their questionnaires whether they had capacity or not. They also highlighted the importance of contemporaneously recording the data collection methods used at each timepoint because in these populations the person who was providing data might change over time.

And just recording that whole process - being able to record who’s actually completing that questionnaire, is it a proxy or not? And making sure that we have those at all of the different time points, as it might not be the same for the whole trial. [ID 05, trial statistician]

### Importance of having access to support and resources

#### Lack of access to relevant expertise

Where trial teams did not have relevant internal experience, the lack of access to external expertise was reported to be a considerable barrier. Some participants described the difference that having a knowledgeable ‘colleague down the corridor’ would make if available, and this lack of access to expertise ultimately impacted on their confidence and ability to design trials that included adults lacking capacity.

We did not have any particular expertise in our research team and institute, you know someone who can advise us on okay, if you want to involve people who lack capacity this is what you should do, this is how you need to write the ethics approval, this is how you should just carry it out. So, we just had no idea how to do it. [ID 02, care home researcher]

Participants also expressed a desire to have input from sources of expertise such as the HRA when they were navigating the contextualised intricacies of designing trials involving adults lacking capacity against a backdrop of the complexities of the legal frameworks. Analogies were drawn with the ability to consult the Medicines and Healthcare products Regulatory Agency (MHRA) for scientific advice at any stage during the development of a CTIMP trial [43]. Some participants reported the impact of not being able to seek advice from ‘flagged’ NHS REC with expertise in reviewing research involving adults who lack capacity ahead of submitting their application for ethics review as their input was only accessible once an application had been submitted. Other participants described how trials that excluded adults who lacked capacity would be reviewed by a REC that was not necessarily experienced in reviewing research involving adults lacking capacity and therefore they were unable to benefit in terms of potentially increasing the inclusivity in their current trial or to inform their design of future trials.

#### Resource-intensive nature of trials including adults with impaired capacity

Trial setup was frequently reported to be prolonged for trials involving adults lacking capacity due to additional delays associated with preparing alternative forms of documents and for gaining ethical approvals. In addition to standard participant information sheets (PIS) and consent forms, trials including adults lacking capacity had to create versions of the PIS for consultees and legal representatives, and an alternative consent form or consultee declaration form. Some trials used the same version for both personal and nominated/professional consultees and legal representatives, whilst others developed separate versions. Some trials also created accessible information for participants with cognitive impairments (e.g. brief summary, pictorial or easy-read versions, video format). The development and document control aspects of multiple documents required additional resources, particularly to ensure that effective public involvement was undertaken, and where versions translated into other languages were also being used.

[A trial] where maybe you need only two information sheets, it is quicker. But for this, I think last time we probably submitted about ten or twelve different information sheets, and you want to consult with different people, you want more time to consult with not just a lay group, you probably want to consult with [care home] residents and with staff and with relatives. So, there’s a lot of work to do to do it properly I think which we’d probably skimp on because we just don’t have the time. [ID 06, trial manager]

However, it was the additional delays around ethical approval, particularly where there were sites in Scotland as well as England and Wales that was reported to have the greatest impact. Again, this was adversely affected by a lack of expertise in conducting these trials.

We have tried to maintain the same protocol across both nations because I thought at the outset that that might be easier. With hindsight it probably wasn’t but the advice wasn’t there when we set off, so we had to just do what we felt would work. Essentially, we've got a different IRAS number for the capacity elements of the study in Scotland and not the rest of it, and we almost have two complete document sets now. [ID 17, trial manager]

The dual ethical approval processes meant that applications were reviewed sequentially—in England and Wales and then Scotland where only one NHS REC reviews research involving adults who lack capacity—which impacted on opening sites and potentially having to re-submit documents where one REC required changes but not the other.

So that in itself was quite tricky because for a while we had ethical approval in one country, but we didn’t have it in the other and then if we had any amendments they had to go separately and then we had to wait. [ID 20, senior trial manager]

Differences included terminology and who could act as an alternative decision-maker, as well as processes of establishing if a participant lacked capacity to consent. For example, in Scotland, certificates of incapacity are issued under the AWI [25], and so protocols and context-specific site training needed to take account of these differences. Some trials reported having very high numbers of different consent and information documents. Where trials involved emergency situations, it became even more complex, with accompanying additional uncertainties over timescales.

Scotland have slightly different guidelines again, so we’ve had to kind of model pathways that are as universal as possible but representing the differences. But obviously, there is the worry that they may disagree with our process, in which case you’ve then got to go back and potentially start that process again, you know, in terms of protocol and so on. [ID 22, senior trial manager]

It also led to knock on delays such as database development which was already complex due to the array of forms required, multiple addresses (participant, consultee) to be stored, and potential changes to data collection (self-report, proxy completed) over time.

The additional resources needed to recruit adults with cognitive impairments were also highlighted by participants. This included the additional time needed to provide information about the trial to the person with the cognitive impairment, and to check their understanding which in turn might lead to the need for an assessment of their capacity to consent.

It’s knowing that sometimes you have to take time with patients to be able to absorb the information. So, it’s not always a straightforward you can go and complete a consent form with someone. It might be that you go back and forth a few times to allow them to retain the information that they need about the trial. I mean it’s expected but sometimes, you know, we are under pressure to recruit a lot of the time. [ID 16, research team lead]

Assessing whether a participant had capacity to consent to the trial required additional time and also necessitated having trained and experienced personnel to appropriately conduct assessments. This was viewed as particularly challenging in populations where capacity fluctuated and for interventional trials which required complex information to be understood. Only a few participants reported having a specific process to follow in a trial. Some described the use of cognitive tests such as Montreal Cognitive Assessment (MoCA) or Mini-Mental State Examination (MMSE) to inform their capacity assessment when the test was already being used in the trial, or applying the principles of the MCA to conduct an assessment. Although some reported the challenges of assessing capacity.

So, I found it very difficult, if not impossible, for me, as a researcher, to be able to assess their capacity. I mean I’ve never done any kind of formal mini mental state exams or anything like that. Any kind of formal capacity assessment other than following the principles of the Act… so I go by those principles rather a kind of battery of tests which I know that other people have done. [ID 02, care home researcher]

Others described using the judgement of colleagues who may have been more experienced in assessing capacity or with this population, or the person’s usual care team who may have been more familiar with the person, which all took additional time. As the assessment was often recorded on study-specific forms in addition to the person’s clinical care records, additional trial documents were needed.

Having assessed a potential participant as lacking capacity to consent, recruiting staff then needed to identify and approach someone to act as a personal consultee or legal representative. This was considered to be the most resource-intensive part of recruiting adults lacking capacity. In non-emergency trials recruiting in secondary care settings this was generally more straightforward, although it may still have required multiple attempts to contact a family member. However, it was especially time-consuming in settings where family members may not be available or are not in close contact such as in care homes, or where there was little way of contacting families such as remote recruitment in primary care. Even once a family member had been identified, there were issues around non-responses that led to uncertainties about whether the family member had received the information, was not willing to act as consultee or legal representative, or if this should be interpreted as a passive form of declining participation on the person’s behalf. This led to potential participants being left in a ‘state of limbo’.

I may have gone out [to the potential participant’s home] and realised that that person may need a personal consultee, but there wasn’t anyone that they lived with, a spouse or anyone, or a family member in the home. So, I would then often leave the personal consultee information and I would write my name and number on it and sometimes I would get contacted, other times I wouldn’t. But then you don’t know whether that information’s been seen, or acted on, or whatever. [ID 08, researcher in older people]

If no family member or close friend could be identified, an additional process was needed to identify and approach someone in a professional capacity to act as a consultee or legal representative. This could be a member of the person’s usual care team, e.g. the doctor responsible for their care or a member of care home staff, provided they were not involved in the trial.

We kind of go down the route of trying to identify a [care home resident’s] family member or friend to start with, and in most cases, there would be somebody that we can send information to initially. We would send information out to them, give them a couple of weeks to contact us or to send back their agreement form, and then we’d send out a chase, and then if we hadn’t heard anything another seven days after that, then we’d go down the route of identifying a nominated consultee. [ID 06, trial manager]

In some care homes, a suitable member of the care team would be identified once a consultee or legal representative was needed, in others it was a pre-delegated and one member of the care team would take on this role for all residents if needed. Not all care homes or staff were comfortable with this process however, and some required additional explanation and reassurance or did not wish to use this approach in their care home. In secondary care settings, some research nurses reported having prospectively created a pool of colleagues to act as potential consultees or legal representatives which facilitated the process. This sometimes took the form of a log that was maintained by the research nurse and was considered particularly useful in time-sensitive trials such as those involving trauma. It was viewed by some as a way for clinical staff to get involved in supporting research without the requirements of GCP training. It was reported that some felt reassured that it was an interim arrangement as consent would later be sought from the patient or their family, although there was no guidance or support for this role in any of the trials.

They know that it’s only a finite consent that they’re giving because the next step will be either the participant themselves if they agree [when] gaining capacity, or the relative. And the professional consultee [sic] only stands if the patient dies. [ID 13, research nurse]

Participants also acknowledged the additional time required for collecting data and completing outcome measures both with participants with cognitive impairment and when proxy reported.

Obviously a lot of them are having to sit down and fill out measures with staff, it’s not just handing out, you know, if you were doing a trial in an adult population, or something, you just give them a load of questionnaires to fill out themselves and it just doesn’t work like that for studies where you’ve got to get staff to do proxy measures, or you’re getting self-report from people with dementia, you’re having to sit down with them to do that. [ID 15, Chief Investigator]

These resource implications and the need to meet expected or metric-driven recruitment rates sometimes resulted in participants feeling under pressure to recruit more quickly or to find alternatives to including participants with impaired capacity.

Oh, you’ve only recruited such and such an amount? Like what’s the delay can you not just move it on? I think with this population things take longer, and recruitment takes longer, there’s more people involved, there’s extra things to be sensitive, mindful about. Yeah, wouldn’t it be easier if we could just talk to their parents or talk to their support workers and get their answers, but just because something’s easy doesn’t mean it should be that way. [ID 03, learning disabilities researcher]

Having adequate support and resources was seen as a key facilitator, and some participants described how their experiences had led them to build in additional resources when developing funding applications, beyond those usually available through R&D departments or clinical research networks.

We funded a half–time research nurse at every site because of the difficulties in working with these patients and their families, and the sorts of visits that were needed, going out to their homes. It was quite intensive so that was funded on the grant. [ID 20, senior trial manager]

However, the competitiveness of funding applications that need to be seen as offering value for money alongside other trials with populations which are more straightforward to recruit was a significant challenge. Participants also reported the challenges of having caps for particular funding schemes, e.g. the tiers in NIHR Research for Social Care (RfSC) and Research for Patient Benefit (RfPB) programmes, which did not take account of these additional resource requirements. This was particularly the case where the research was conducted in non-NHS settings such as care homes and so did not have equitable access to research delivery infrastructure.

The challenges of pressure to conduct more inclusive trials, with limited funding and resources available to adequately support inclusion, were also highlighted as a barrier to both conducting trials involving adults lacking capacity and other under-served populations in general.

Sometimes you have to bear in mind you need to get the numbers, but you also need to think about inclusivity with that …. I don’t think resources is the reason not to do it, but it’s got to be a consideration. You know, you can’t ask for the world and not fund it. [ID 12, qualitative researcher]

The role of funders and grant reviewers in encouraging inclusive research was welcomed; however, the responsibility to conduct more inclusive trials was viewed as being shared between funders, RECs and research teams.

Applications where you force people to think about how [they] have they addressed inclusivity and then also being willing to provide funds to support those extra [requirements], because it does take time and the cost in some cases is huge. I think making researchers think about it earlier, but funders also being prepared to allocate resources to support that I think is key. [ID 25, senior trial manager]

#### Effective communication processes

Participants described the role of effective communication in successfully conducting trials involving adults lacking capacity. This included communication between members of trial teams when designing and co-ordinating trials, but also the importance of two-way communication between the trial team and those delivering the trial at research sites to ensure that key messages about the barriers being encountered and strategies to overcome them were being fed back. It also included communication between clinical staff and research staff at sites to more closely integrate research and clinical care and to ensure that potential participants and their consultees and legal representatives were being identified and communicated with.

We’ve got quite close links with the Trauma Coordinators, they’d be like I’ve already spoken to [the patient’s] next of kin, and she’s happy, here’s her number, and what have you. It’s about communicating as a whole team and to discuss it with colleagues and trial offices about the problems and things that were working well and things that weren’t. [ID 18, research nurse]

Communicating with potential participants with cognitive impairment was viewed by research nurses and others involved in recruitment as requiring confidence and experience, as well as having accessible information to facilitate information provision. This included the use of summary information sheets as well as alternative formats which could be used to scaffold information about the trial and enable to person to express their informed views about participation where possible.

We did actually have an aphasia friendly information leaflet for those who didn’t have capacity [to consent] but had some capacity to understand the study. It was a large font, so it was fourteen point, one and a half times spacing, the layout was in boxes with images, so that was all broken down. [ID 19, trial manager]

Other strategies to overcome communication difficulties included approaching the person when family members were present who could support the person to understand the information and make a decision about participation where possible, or the involvement of interpreters where language was considered to be a barrier. Where research nurses considered a communication disorder to play a role in capacity and decision-making, particularly for people who had experienced a stroke, some reported seeking the involvement of a speech and language therapist (SLT) or tagging their discussion about the trial onto the end of a routine SLT session where possible.

Effective communication was also viewed as an important part of engaging with family members who were approached to act as a consultee or legal representative and was considered to be particularly challenging when the approach was made remotely, e.g. via post, or when usual care teams had to be relied upon to send out information about the trial due to data protection requirements.

You don’t want to harass the relative. So, unless the relative contacts us, we can’t contact them. So, unless the relative says, I would like some more information about this, we can’t give them more information even though we’ve written to them. I naively said, ‘Well why can’t we ring them up a few days later, and check they’ve got the letter and ask them what they thought about it?’, but you can’t do that. [ID 11, Chief Investigator]

The challenges of family members making a decision about participation on the person’s behalf was also seen as a barrier, primarily around how families can be orientated to base their decision on the person’s wishes and feelings rather than their own views, and how they might access those wishes in the absence of explicit discussions about research participation.

I think it’s hard to try and say what that person would want if you haven’t specifically had that conversation with them previously about whether they’d want to be involved in research or even what types of research. It’s difficult when you’re asking someone to consent on behalf of someone else, they are always going to have their own views, and it’s difficult with the best will in the world, to make a decision based purely on what someone else would want and not what you would want. [ID 01, research governance lead]

Building a rapport with family members and using a person-centred approach to discuss what mattered most to the person was viewed as a useful approach to supporting the family member to make a decision based on the person’s wishes and feelings.

Issues around communication were also raised when there were changes in a person’s capacity status during a trial and consent was revisited. This including the use of a ‘deferred consent’ model where the person was enrolled without prior consent (or through brief verbal consent) during an emergency and then consent to remain in the trial was sought from them once they had recovered or their family approached if not [44]. In these situations, they had received the intervention at the point they were approached, and so they were informed about their participation and were asked to provide consent for data collection to continue. Participants recognised the sensitive and potentially challenging nature of these discussions, and the need to tailor the timing and approach to the context and the person’s ongoing health needs. Research nurses highlighted the importance of maintaining a relationship with the person, their family and the care team throughout their time in a study, including regular bedside conversations when completing visits to the ward for data collection. This also provided opportunities for ongoing assessment for any changes in capacity. Participants reported rarely encountering anyone who declined to continue in a trial, with high levels of acceptability towards the consent approach used, although there were instances of people being discharged or transferred or dying before consent could be sought.

At the time we get to them all the work has been done, so it’s just asking consent to keep the data and I’m always surprised at how accepting people are. It’s very rare that we have somebody that is really unhappy about what has happened, once you sit down and explain to them. And many patients are really happy that they’ve been able to contribute to improve treatment for somebody like them in the future. [ID 16, research team lead]

Good communication with RECs was also seen as facilitative, and research teams described how building a relationship with particular RECs meant that there was a shared understanding about the ethical issues involved and how trials could best be designed to overcome these. However, this supportive communication with RECs was not experienced by all researchers, with some expressing frustration with the process and the inability to discuss these complex applications in detail or in advance of submitting an application.

One of the panels didn’t seem to get it at all, and it was like really difficult to try and explain. The other problem is that when panel’s running behind they might not allow enough time and so when [the researchers] got into the room, they’ve got like five minutes and so they didn’t get a chance to answer, be asked or answer any of the questions or explain anything, so it was all very rushed, and not having a chance to actually talk through the study and have a conversation about it. [ID 15, Chief Investigator]

What would have been useful, and I don’t know whether it is accessible, is contacting REC beforehand to kind of talk through that proposal because, obviously, if we had had that conversation and they’d gone, well, you need to make these changes. Well, that could have just pretty much saved us probably two and a half, three months. That’s a massive amount of time. [ID 22, senior trial manager]

#### Use of adaptive strategies including COVID-19-related adaptations

The importance of designing in flexibility in a trial, and adapting processes in response to challenges encountered, was seen as key to the successful delivery of trials with this population, including in response to the pandemic. Participants expressed how COVID-19 had presented particular challenges which highlighted the need for adaptive strategies in response to the high numbers of acutely and critically ill patients, the introduction of Urgent Public Health studies with a pause in almost all non-COVID trials, and universal infection control measures. Challenges also included the involvement of research-naïve and redeployed staff who required rapid general research and trial-specific training that was sufficiently comprehensive but minimally burdensome. However, the greatest impact was the stopping of all hospital visitors which included family members who would act as consultees and legal representatives. Participants reported a change in many trials towards enabling remote forms of consultation with families through email, phone calls and video calling which facilitated the recruitment of people who lacked capacity to consent. This included COVID-19 trials as well as amendments to non-COVID trials in other settings such as care homes and the community. COVID-19 was seen by some as a driver in enabling long-awaited advancements in trial conduct.

I think there was question about whether that [the use of professionals as nominated consultees] could happen, but since COVID that has now come in as being acceptable and they’ve had a substantial amendment on the trial to allow for professional consultee [sic] to be enough. [ID 13, research nurse]

Research nurses talked about the value of sustaining this flexible approach and embedding it in more trials in the future that recruit adults lacking capacity.

It would be better to have a few more [studies allowing] consent that we could do over the phone with relatives. Because it’s something that we’ve found to be quite helpful to have with some of the COVID trials and they’ve adapted some of the non-COVID trials as well. But many of our trials don’t have that at all. [ID 16, research team lead]

However, others reported that the challenges of communicating about the trial with these populations and those who care for them may not be easily addressed through these adaptions which limit personal interaction and the building of relationships.

In the original study, we were going into the [care] home. We were going to meet people face to face and now we’re trying to do that remotely via Zoom, so it’s sort of turned into a different animal. In a way that also brings subtle changes to the way you engage with people. Very, very different. [ID 11, Chief Investigator]

Research nurses described the challenges of communicating about a trial with patients with COVID-19 who often had impaired capacity due to hypoxia or other conditions. This was intensified by the requirement for research staff to use PPE (personal protective equipment) and reduce physical contact which limited non-verbal communication, and patients requiring respiratory support such as CPAP (continuous positive airway pressure) hoods which made it difficult for them to hear above the noise and meant that patients who usually wore glasses may not have been able to read information about the trial. Even research nurses who were familiar with impaired and fluctuating capacity in a range of conditions described the more complex nature of assessing hypoxia-related capacity impairment. Other COVID-19-related challenges included the infection control risk from contaminated paper consent forms which necessitated alternative approaches such as photographing or quarantining completed forms, or the use of witnessed verbal consent or e-consent with people with capacity as well as consultees/legal representatives for those who lacked capacity. However, the use of digital tools such as email and video conferencing raised additional concerns about the exclusion of those without access to such technology.

Outside of secondary care, challenges included care homes closing to all non-essential visitors which included research staff, and ‘lockdown’ restrictions which prevented research visits in primary care. For trials that remained open, this necessitated a rapid change in contact methods with potential participants, their families, and sites such as care homes, which could also be affected by limited digital access and poor connectivity issues. It also presented new challenges such as having to undertake virtual assessments of capacity which were unfamiliar to researchers.

However, research nurses reported a positive impact from COVID-19 where the high-profile and urgent search for effective treatments for this novel disease meant that there was a greater awareness about research in the general public. This facilitated discussions about trial participation with both patients and their families when contacted. COVID-19 adaptations also removed geographical location as a barrier to participation in some instances and led to greater embracing of digital technologies for some groups. This was seen in a study involving people with learning disabilities which originally recruited through local advocacy groups but switched to online recruitment and data collection.

So originally, because it was meant to be face-to-face around [local area], when they went on-line it was actually an opportunity because they were recruiting people from all over. There’s people who weren’t involved in any of the advocacy groups prior to the pandemic, but then they’d been looking for like social lifelines and they are now [participating]. [ID 03, learning disabilities researcher]

### Need for building a body of knowledge and expertise to support future trials

Some participants identified the need to build research capacity that could support future trials in populations affected by impaired capacity to consent and in the settings where they received care. This included greater support for clinical and non-clinical academic careers in relevant areas/conditions and ensuring parity of research support and incentives for non-NHS settings to participate in research. Given the complexity of these challenges, participants described a need for sharing collective experiences and ‘lessons learned’ to inform the design and conduct of future trials, as well as ensuring better training and support for those involved.

#### Need for enhanced training and guidance

Whilst many participants referred to having received generic Good Clinical Practice (GCP) training, this was usually in the context of its inadequacy to prepare researchers for conducting research with adults with impaired capacity to consent. This included a lack of information about the complexities of the legal frameworks, failing to highlight population and context-specific issues, as well as the view that it was not designed to apply to non-CTIMPs or social care research which limited its usefulness.

I thought it [GCP] was a bit lacking, because it is very brief around the research process, but I don’t think there was enough of that distinction between capacity to consent and capacity to take part in the intervention. I don’t think GCP covers it enough, or if it does it’s been lost on me. I think they should split GCP into CTIMPS and non-CTIMPS studies. [ID 04, research programme manager]

Some participants had received other training specifically on adults lacking capacity, such as modules offered by the NIHR, but emphasised the need for more context-specific and detailed training. They particularly emphasised the need to link training with practice and for an interactive component.

I did the ‘recruiting patients who lacked capacity’ NIHR training, but I think until you actually kind of do it hands on….. So, kind of, talking with other people about it, I think more would be useful. [ID 21, trial manager]

Participants also reported the need to extend the training to include mental capacity assessment specific to capacity to consent to research. One participant suggested that videos of an assessment being conducted could be helpful, and another suggested that training was most needed for non-NHS studies or by non-clinical researchers.

I did go on an adults lacking capacity course from the [name of research network] which was not really fit for purpose so the training that does exist needs to be completely rethought. They need to give researchers a practical grounding in how do you assess capacity. Not necessarily in an NHS sited study, you know how do you do it in a social care? There’s more and more focus now on social care research but the support for it in terms of capacity to consent and the training for that hasn’t caught up. [ID 02, care home researcher]

Some members of research teams expressed uncertainties about the process for assessing capacity, with some describing problematic processes in some trials such as requiring blanket assessments regardless of the legal presumption of capacity under the MCA.

And they had to do that for every participant. Which I had a few questions about in the early days, because some people were saying, “Should capacity not be assumed unless thought otherwise?” But, because of the population, we needed to be clear on whether they did have capacity to consent or not. So, they were all assessed at the outset by the nurse taking consent. [ID 20, senior trial manager]

This supports participants’ suggestions that there is also a need for wider training around capacity and consent for researchers, REC members and funding panels. Some participants called for more context-specific training and guidance for certain groups such as care home staff or ED staff, and for trial managers who often play a central role despite sometimes having little training or experience in this area.

From a trial manager’s perspective, I think support and guidance in doing that particularly around correct terminology for who needs to give consent, and how it varies between the different countries, and that sort of thing to give people more confidence to set up this sort of study. And also, because the nurses will quite often contact the trial team when they’ve got a tricky consent question, to run it past you. [ID 20, senior trial manager]

In addition to training, participants described a need for improved information and resources around trials involving adults lacking capacity, beyond existing provision such as from the HRA.

I’ve been stabbing in the dark like ‘where should I look for reliable information’? A lot of it was potluck Googling ‘oh, fabulous, that’s something I can understand’. Don’t get me wrong, the HRA website became a bit of a go-to because they’ve got that decision tool page. But, despite the fact that we reviewed that website, it still took the thrashing at the REC meeting for them to go well, no, actually, you’ve not interpreted that correctly or that you’ve not thought about this. [ID 22, senior trial manager]

Participants were also asked for their views about a number of resources currently being developed by members of the research team to help researchers when designing and conducting trials involving adults with impaired capacity. This included an online toolkit which collates resources on capacity and consent to research, such as guidance around the legal frameworks, assessing capacity and developing accessible information, and how you identify the appropriate person who should be consulted or give consent [45]. Some participants were already aware of the website and described its usefulness, others emphasised the importance of disseminating information about it as part of its implementation and linking it to other tools such as the HRA website or IRAS forms. Participants also suggested that its value could be increased by adding case studies or including a discussion forum where questions could be posed and answers ‘crowdsourced’.

Other resources in development include a framework to aid researchers when designing trials involving adults with impaired capacity. The framework is being developed as part of the NIHR INCLUDE’s work around inclusivity of under-served populations [11] and follows on from the development of the Ethnicity Framework [46]. Whilst cautioning about over-burdening researchers, a practical framework was widely welcomed by participants who described it as potentially ‘enormously helpful’ and expressed urgency about the need for more support.

I think that’s a fantastic idea. Of course, patients lacking capacity are excluded from studies and I see a lot of people get to that box [on the IRAS form] and just say ‘no’. Well, why if we have equipoise and there may be benefits to the patients then not having capacity should never be a barrier. I don’t understand … so definitely I’d be fully in support of that. Please quick, quick, we need it. [ID 07, Clinical Trials Unit manager]

Participants also stressed the need for any training and guidance being developed to be ‘joined up’ in order to provide a comprehensive, coherent and consistent message.

#### Developing future interventions

The need for the development of interventions to support trials involving adults lacking capacity was also highlighted by participants. At an individual level, this included better ways of supporting consultees or legal representatives’ to make informed decisions about participation through building on a decision support intervention that has been previously developed and is currently being evaluated with families [36]. This could take the form of developing a multi-media version of the decision aid, or developing other interventions to reduce the burden on families or creating an equivalent decision aid for professionals acting as nominated consultees or professional legal representatives. It could also include gaining a better understanding about consultees or legal representatives’ decisions to decline participation on the person’s behalf, or to hear their experiences of being approached.

One thing I did think about was a kind of screening process to record reasons for not participating [from] consultees, which I don’t think we really fully do at the moment. So that we can capture those reasons, and then think about if we need to address things in the trial. [ID 05, trial statistician]

I think I would’ve been given a lot more confidence if I had actually heard from two or three different relatives, about how they were approached, how they felt about it, and actually be given the other side of the coin because I've never seen that. [ID 13, research nurse]

Participants also suggested that more tools to support people with impaired capacity to make an informed decision about research participation such as videos or simplified consent forms would also be useful.

At a policy level, suggestions included streamlining the process for gaining ethical approval for trials involving adults lacking capacity, particularly where two different countries were involved. Changes to the legal frameworks governing advance planning for research, and the development of interventions to support people to prospectively express their wishes about research in the event of losing capacity to consent were also supported by participants. This could be embedded into care pathways or at the point of registering for services.

I think it would be incredibly helpful, especially for dementia. It might not be a conversation to have at the point of diagnosis because people are still taking that all in but yeah, at some point while people still have capacity it would be really useful to have that conversation. With mental health conditions as well, things like schizophrenia, if you’ve got someone who’s relatively stable and well and you could ask them a question, you know ‘if you became unwell and there was an opportunity for a new treatment, would you be interested in it or not?’ [ID 01, research governance lead]

## Discussion

Researchers and healthcare professionals involved in the design and conduct of trials with adults with impaired capacity describe a circular paradox where trials including adults lacking capacity to consent are relatively uncommon; therefore, they have less experience and confidence in conducting trials with these populations and so are less likely to design trials to be inclusive of these populations. Challenges are encountered at every stage throughout the lifecycle of a trial; however, they are primarily clustered around early trial design decisions, when navigating ethical approval processes, and the recruitment and retention of participants with impaired capacity. Whilst some of the barriers and facilitators described by participants are context specific, some are more universal including the need for better training, guidance and interventions to support the design and conduct of future trials.

The context-specific challenges identified in this study reflect those encountered in numerous previous studies exploring research in settings such as critical care [47, 48] and care homes [49], and the issues surrounding capacity and consent in populations such as those receiving palliative and end of life care [50], people with aphasia [51] and people with learning disabilities and/or autism [52]. Our findings that highlight a universal knowledge deficit are also supported by previous studies which have examined understanding of the legal frameworks governing research involving adults who lack capacity by health and social care professionals [26], RECs [53] and researchers [54, 55]. It is also supported by our previous study which analysed study information sheets provided to consultees and legal representatives and found that many of the documents (which had been designed by researchers and approved by RECs) contained inaccuracies about the legal arrangements and some misinterpreted the legal frameworks [27]. Similarly, we have previously highlighted issues around gatekeeping in research involving adults lacking capacity [56]; however, this study provides further evidence to support this and identifies areas or settings where this occurs. Of note is the role social workers may play in gatekeeping although their involvement in research decisions is not in line with the arrangements laid out in the legal frameworks.

This study also echoes the previous calls for more detailed guidance in relation to the emerging uncertainties around the operationalising of the MCA in research practice, and for researchers to share their experiences of working with the MCA [57]. It also supports the calls for RECs to have greater expertise in alternative consent processes, such as those required in emergency and critical care research, and to provide greater guidance on these to researchers [47]. However, this study extends this lacuna in knowledge to identify which groups would most benefit from more guidance and training and explore what content and format these might take. We also identified a number of target areas for the development of supportive interventions, including for families and professionals acting as consultees and legal representatives, as well as for people with cognitive impairment and those who may lose capacity in the future.

The need to adapt trials during COVID-19 to reflect the infection control challenges around obtaining and documenting consent, including from legally authorised representatives of COVID-19 patients, is supported by other studies which have explored these barriers and identified the resource implications [58]. However, previous studies have not explored this in a UK context, nor in trials outside secondary care settings where there are additional challenges due to restrictions on researchers visiting. COVID-19-related changes in working arrangements for researchers may also have impacted on the ability to access and learn from the knowledgeable ‘colleague down the corridor’, or to informally share information between teams and team members. Aside from COVID-19, the need to adapt qualitative research methods for populations with cognitive impairment has been well described in fields such as dementia research [59], but are lacking when it comes to embedding qualitative research in trials which has focussed to date on participants with the ability to provide consent for themselves. Our findings about the important role public involvement plays in trials involving people with impaired capacity is supported by a number of studies demonstrating the impact of public involvement in trials generally [60].

In terms of the resource-intensive nature of these trials, the additional costs required to recruit people with cognitive impairment has previously been highlighted in a community-based trial, as well as the problematic nature of cost attribution in these trials and the lack of evidence on which to base the justification for additional research activity costings [61]. However, these issues affect all trials involving adults lacking capacity and the additional resource requirements must now be acknowledged and met by funders who are committed to ensuring that the research they fund is truly inclusive of under-served populations, such as those with cognitive disabilities [62].

### Strengths and limitations

A key strength of this study is that we systematically explored the barriers and facilitators to conducting trials involving adults lacking capacity across a range of trial types and settings, as opposed to exploring experiences in single trials. This has enabled us to take a wider perspective on the challenges encountered by those who design and those who deliver such trials, thus also highlighting the transferable learning from research practice involving these different conditions and populations. However, whilst maximum variation purposive sampling was used to gather both positive and negative experiences in a broad range of trial populations and contexts, self-selection biases may have occurred. Whilst the participants had experience of conducting trials across the UK and internationally, they were based in England and Scotland and so the barriers and facilitators reported may be less applicable in other countries with different legal frameworks and infrastructures to support trial delivery. However, some challenges such as the complexity of the legal frameworks and ethical issues involved may be more universal in nature [63]. We did not include the experiences and views of people affected by conditions that impair their capacity to consent as this was beyond the scope of this study.

It is also recognised that inclusivity of under-served populations in trials, such as adults lacking capacity, does not begin and end with the activities described in this study but covers the whole lifecycle of a trial from identifying research priorities to dissemination and beyond [11]. These vitally important public involvement and engagement activities also encounter challenges when involving people with cognitive impairment and so require further exploration. There are many useful examples of successful approaches to public involvement to consider, including the involvement of people living with dementia [64], aphasia [65], and in mental health and learning disability research [66]. It is also important to recognise that the inclusion of groups who are under-served by research through virtue of a cognitive disability may also be impacted by other intersecting factors such as ethnicity and being socio-economically disadvantaged. Whilst only minimal demographic data was collected for participants in this study, these groups are less likely to be included in trials methodology research and so trials may be designed in a way that fails to take account of their views and experiences, including the acceptability of alternative consent models such as deferred consent [67].

This study focussed on trials rather than research more broadly as trials are considered more challenging to recruit to as they require greater commitment from the participants in terms of time and risk than other types of studies [68]. Therefore, other important aspects of enabling and supporting people with cognitive impairment to participate in research, such as through the use of participatory action research and/or creative research methods, did not arise in this study but are recognised as having a valuable part to play in ensuring equality of opportunity to participate in research.

### Recommendations for research practice and future research

We have made a number of practice recommendations for specific stakeholder groups (see Table 4), including funders of health and care research, RECs, leads with responsibility in the areas of policy, governance, and infrastructure, trialists and teams who design and conduct trials, and research staff who recruit participants. These should be considered alongside more specific recommendations for recruiting adults with impaired mental capacity at the end of life in research from the MORECare Capacity project [50].

**Table 4:** Recommendations for addressing the barriers to conducting trials with adults with impaired capacity to consent

Recommendations by stakeholder group	
**Funders**	
1. Funding committees should consider whether issues around capacity and consent have been appropriately considered by applicants proposing research involving populations where these issues may be encountered. This may include requesting justification for their exclusion if appropriate.	
2. Funders should include signposting to information and guidance on the design and conduct of trials involving adults with impaired capacity to consent for researchers developing applications involving these populations.	
3. Funders should acknowledge the additional resources needed to recruit under-served populations, such as those where capacity may be impaired, and ensure adequate provision for these ‘missing’ costs. This should also be considered when making comparisons between the cost-effectiveness of applications where under-served populations are included.	
4. Funders should ensure appropriate provision is made to support public involvement with people who have a cognitive impairment, beyond generic support for public involvement. Enabling and supporting these groups to access and contribute to public involvement opportunities requires additional resources such as developing appropriately accessible materials and activities, arranging smaller group interactions which may involve multiple short meetings, and additional funding for carers or other forms of support for the person with cognitive impairment.	
5. Funders should take into account that, in addition to these trials being more resource intensive, they are often conducted in care settings with less access to research infrastructure support, such as care homes. The increased resource needs should also be considered in relation to the application of funding ceiling caps or tiers.	
**Research ethics committees (RECs)**	
6. REC members should ensure they are familiar with the ethical requirements for research involving adults lacking capacity and the practical application of the legal frameworks governing the different types of studies (CTIMPs and non-CTIMPs).	
7. When reviewing studies, RECs should consider whether issues around capacity and consent have been appropriately considered by researchers. This applies to all trials not just those explicitly including adults lacking capacity, or just ‘flagged’ RECs. This may include requesting justification for the exclusion of adults who lack capacity rather than only justification for their inclusion as required by the legal frameworks.	
8. RECs should consider whether researchers have appropriate arrangements in place in the event that capacity is lost or may change or fluctuate during a trial, which may include all trials not just those intending to recruit adults lacking capacity.	
9. Enabling consultation and communication between RECs, the HRA, and research teams prior to submission of an application may ensure that any questions or issues are addressed at the earliest opportunity and reduce subsequent delays in applications receiving a favourable opinion.	
**Policy/governance/infrastructure leads**	
10. Organisations with oversight or responsibility for ethical review processes should seek to address inconsistencies in the review of studies involving adults lacking capacity and the quality of the advice provided to researchers.	
11. Research governance and ethical review processes should be harmonised and streamlined across the UK to reduce the impact of a dual REC submission and to enable research involving adults lacking capacity to have equal parity in time taken to review with studies involving people who are able to provide consent.	
12. R&D infrastructure and support should be reformed to take account of the complexities encountered in the delivery of trials involving adults lacking capacity. For example, metrics and associated accruals should be revised to take account of the additional time and resources required to recruit adults lacking capacity.	
13. Co-ordinated and comprehensive training on the fundamental principles underpinning research involving adults lacking capacity to consent should be available to all those who design, review, and conduct these trials, with access to supplementary modules containing context-specific information where appropriate.	
14. System-wide initiatives are needed to build capacity and competence in research involving adults lacking capacity. This requires investment to recruit and retain staff with appropriate skills and experience and support for building long-term relationships. This will ensure that future trials can successfully recruit and retain these populations thereby avoiding research waste, as well as addressing fundamental issues around their exclusion.	
**Trial teams**	
15. Research teams should ensure they have access to methodological expertise and/or input from people with experience of designing and conducting trials involving adults lacking capacity to consent and an understanding of how the legal provisions for adults lacking capacity are implemented in practice.	
16. Flexibility and inclusivity should be ‘designed into’ trials from the outset which may include the use of alternative consent arrangements (e.g. remote consultation with personal consultees and legal representatives via telephone or video conferencing, enabling verbal or electronic consent/agreement) with adaptations made in line with feedback from recruiting sites.	
17. Research teams should consider trial designs that enable the collection of additional data to inform the design and ongoing conduct of the trial, such as early qualitative work with synchronous analysis and feedback to enable changes to consent processes and/or enhanced consent training provided to recruiting staff.	
18. Research teams should ensure that meaningful public involvement is planned and implemented, including supporting people with impairing conditions and their carers to become and remain involved. This may include ensuring that costs for extra care provision are available to carers so that they are able to attend meetings and ensuring that materials and methods used in the public involvement activities are accessible. This will require additional resources and time and should therefore be built into funding applications.	
19. Researchers should include additional resources (e.g. enhanced research nurse time) in funding applications in order to meet the additional requirements to provide tailored information and support to participants with impaired capacity, assess capacity if indicated, and identify and approach consultees or legal representatives if required. Resources to support revisiting of consent and capacity throughout the trial will also be required.	
20. Trials that are reliant on remote contact only during a trial (e.g. postal recruitment or follow-up) may need to consider alternative methods of contacting participants and ensuring that data can be collected. This may include recruiting participant-carer dyads and including statements in documents to encourage reporting of any cognitive difficulties and clarify who is completing documents.	
21. Complex and lengthy Participant Information Sheets that are not cognitively or linguistically accessible should be avoided, and researchers should ensure that accessible trial information (e.g. easy-read, pictorial, brief summary version) is available for all trials where cognitive and/or communication difficulties may be encountered, with layering of information as appropriate to the person’s needs.	
22. Trials should explicitly include processes for assessing capacity (e.g. when and by whom will assessments be conducted, what training and documentation is required), the involvement of consultees and legal representatives with provision of corresponding study documents, arrangements in the event of a change in a participant’s capacity status during a trial, and arrangements for managing data in the event of a participant’s discharge, transfer or death before consent can be sought, for example when using a ‘deferred consent’ model).	
23. As capacity may be lost during a trial, researchers should consider making prospective arrangements for the participant to continue in the trial (if considered appropriate to the trial context), such as including an explicit statement on the consent form. Trials may wish to consider asking the participant to identify a family member or friend who is willing and agrees to be approached to act as consultee or legal representative.	
24. The dissemination of research fundings must be designed in a way that takes account of any context and population-specific barriers to dissemination, for example ensuring summaries are provided in cognitively and linguistically accessible formats. Alternative and purposive dissemination pathways may be needed to ensure that information about the findings reach participants and their carers, and may need to ‘mirror’ recruitment arrangements.	
25. As part of reporting trials, researchers should detail the consent model and recruitment approach used and describe the trial population included (e.g. proportion who lacked capacity to consent), to ensure the results are viewed in the context of the representativeness of the trial. Any challenges encountered and lessons learned to overcome these should also be shared in order to create community-sourced evidence about the effectiveness of different strategies that can inform future trials.	
**Research staff who recruit participants**	
26. Additional training and support may benefit those recruiting adults with impaired capacity to ensure they have appropriate skills and confidence to involve these populations. This may include ways to enhance communication about trials to people with cognitive impairments and support their decision-making, assess capacity where required (including remote assessment of capacity which may be more challenging), appropriately revisit consent as required, and optimise strategies to approach family members to act as consultee or legal representative.	
27. Staff who are less familiar with populations or contexts where there may be particular challenges around capacity to consent may benefit from peer support or the opportunity to shadow colleagues with greater experience with these populations. This might include learning from approaches used in speech and language therapy (SLT), with specialist input by SLT where required.	
28. Involving family members and usual carers at an early stage will help to identify any additional communication or support needs the potential participant might have, including language requirements, and their involvement will help to support the person with cognitive impairment to make a decision about research participation. Research staff should consider including family members in discussions about research where appropriate.	
29. Prospectively planning and having clear processes in place for involving professionals as consultees or legal representatives (if required/permitted) may reduce unnecessary delays in the process. This might be particularly important for trials in emergency settings. This may include creating a list of clinical care team members who are able and willing to be approached to act as nominated consultee or professional legal representative, with appropriate information being available.	
30 Staff should ensure that there is regular communication with the participant’s care team and/or contact with the participant so that any changes in capacity are recognised in a timely manner, and that consent and consultation can be revisited appropriately.	

In addition, recommendations for future research have been identified from this study and are supported by the findings from previous research. These have been mapped below against the areas identified by NIHR INCLUDE initiative as targets for future work to increase inclusivity of under-served populations [11].

#### Funder and regulatory landscape

Funders of health and care research, such as the NIHR, have made a clear commitment to addressing inclusivity in research [62]. However, further research is needed to explore the resource implications for trials involving adults lacking capacity and the ‘missing costs’ that would be required to fully resource inclusivity in this context. This includes the additional costs and resources required for meaningful public involvement with groups where the default group meetings and email discussions are less appropriate due to individuals’ higher communication and support needs. Measures should also be taken to address the complexity of the legal frameworks governing trials involving adults lacking capacity [15], including the discrepancies between the provisions for emergency non-CTIMP research in Scotland.

#### Communications and training

Research is needed to explore the knowledge deficits and training requirements of the different groups responsible for the funding and approval of trials involving adults lacking capacity, as well as those involved in the design, conduct and delivery of these trials. A comprehensive evidence-based training programme can then be developed to specifically target these gaps. However, other challenges such as stakeholders’ attitudes towards involving people with impaired capacity in trials may be harder to overcome, and further research is needed into the underlying attitudinal and behavioural factors that affect the inclusion of adults lacking capacity in research [26].

#### Infrastructure, people and processes

A systems-wide approach is needed to explore how the UK research infrastructure supports trials involving adults with impaired capacity to consent. This should include investigating whether the guidance available from the HRA and other sources enables researchers to design inclusive trials, identify what methodological and specialist expertise research teams have access to and what additional opportunities could be provided to enable them to apply the principles in practice, and to review REC practices to ensure accuracy and consistency in their review of trials involving adults lacking capacity. This could build on ongoing work by the HRA as part of the ‘Think Ethics’ initiative [69] with a particular focus on these ethically complex applications, as well as updating a previous analysis of REC reviews and decision outcome letters [53] to assess whether compliance and coherence with the legal frameworks governing research involving adults lacking capacity is improving. The need for greater communication between researchers and RECs can also be seen as an opportunity to address the wider issue that ethical review processes have a tendency to be viewed as transactional (‘approval’ is something that is sought by researchers and given by RECs), rather than relational.

#### Design tools

In addition to the practical INCLUDE framework to help researchers design more inclusive trials previously mentioned, other tools to help design trial are needed. This includes greater development of validated outcome measures that are appropriate for participants with different cognitive and communication disorders, and proxy-completed versions where required. For example, previous studies have identified the need for more intellectual disability-appropriate outcome measures [70] including in mental health. (Re)designing trial materials to be more inclusive using accessibility principles may increase the opportunity for people with cognitive impairment to participate in trials [71]. Additionally, there is a lack of guidance available for professionals acting as consultee or legal representative and a lack of clarity around their role and the basis for their decision which future research should seek to address in collaboration with people with impairing conditions and their families.

## Conclusions

There is a growing focus on ensuring that trials are truly representative of the population affected by the condition under investigation and are inclusive of populations who are currently under-served by research. Adults with impaired capacity to consent are largely excluded from trials, even in conditions and settings where the prevalence of cognitive impairment is high. This study identified a number of barriers and facilitators to designing and conducting trials involving adults who lack capacity to consent. Their inclusion may be influenced by factors such as knowledge deficits, resource limitations and the complexity of such trials which often involve complex ethical issues. Our findings suggest that greater access to training and resources, and the development of supportive interventions and tailored guidance, is urgently needed in order to build capacity in this area and facilitate the successful delivery of trials involving this under-served population.

## Data Availability

The dataset generated and used in this study is available through submission of a data request to the Centre for Trials Research at https://www.cardiff.ac.uk/centre-for-trials-research/about-us/data-requests.

## Abbreviations

CI: Chief Investigator
CTIMP: Clinical trial of an investigational medicinal product
GCP: Good Clinical Practice
HRA: Health Research Authority
MRC-NIHR TMRP: Trials Methodology Research Partnership
NIHR: National Institute for Health and Care Research
REC: Research ethics committee
TM: Trial Manager
STM: Senior Trial Manager
CTUs: Clinical trials units
UKTMN: UK Trial Managers’ Network
